# Screening for Park Access during a Primary Care Social Determinants Screen

**DOI:** 10.3390/ijerph17082777

**Published:** 2020-04-17

**Authors:** Nooshin Razani, Dayna Long, Danielle Hessler, George W. Rutherford, Laura M. Gottlieb

**Affiliations:** 1 UCSF Center for Nature and Health, UCSF Benioff Children’s Hospital Oakland, 5220 Claremont Ave, Oakland, CA 94608, USA; 2 Department of Pediatrics, University of California, 550 16th Street, Box 0110, San Francisco, CA 94143, USA; Dayna.long@ucsf.edu (D.L.); george.rutherford@ucsf.edu (G.W.R.); 3 Department of Epidemiology and Biostatistics, University of California, 550 16th Street, Second Floor, San Francisco, CA 94158, USA; Danielle.Hessler@ucsf.edu; 4 UCSF Center for Health and Community, 3333 California St., Suite 465, Campus Box 0844, San Francisco, CA 94143-0844, USA; laura.gottlieb@ucsf.edu

**Keywords:** pediatrics, social determinants of health, built environment, mental health, stress, park use, urban greenspace, urban nature, poverty, health inequity

## Abstract

While there is evidence that access to nature and parks benefits pediatric health, it is unclear how low-income families living in an urban center acknowledge or prioritize access to parks. Methods: We conducted a study about access to parks by pediatric patients in a health system serving low-income families. Adult caregivers of pediatric patients completed a survey to identify and prioritize unmet social and economic needs, including access to parks. Univariate and multivariate analyses were conducted to explore associations between lack of access to parks and sociodemographic variables. We also explored the extent to which access to parks competed with other needs. Results: The survey was completed by 890 caregivers; 151 (17%) identified “access to green spaces/parks/playgrounds” as an unmet need, compared to 397 (45%) who endorsed “running out of food before you had money or food stamps to buy more”. Being at or below the poverty line doubled the odds (Odds ratio 1.96, 95% CI 1.16–3.31) of lacking access to a park (reference group: above the poverty line), and lacking a high school degree nearly doubled the odds. Thirty-three of the 151 (22%) caregivers who identified access to parks as an unmet need prioritized it as one of three top unmet needs. Families who faced competing needs of housing, food, and employment insecurity were less likely to prioritize park access (*p* < 0.001). Conclusion: Clinical interventions to increase park access would benefit from an understanding of the social and economic adversity faced by patients.

## 1. Introduction

There is a growing recognition within healthcare that access to and quality of care are not enough to produce health [1]. Increasing awareness of the role of parks in shaping health has led to collective and interdisciplinary actions to increase access to these community resources for health. Public parks and recreational resources promote active living, physical and mental health, and overall well-being across diverse communities [2]. Specifically, public parks offer open spaces and facilities such as playgrounds for individuals to participate in physical activity [3], as well as added benefits such as stress reduction, [4] increased social interaction, and social cohesion among neighbors [5,6].

Added benefits of parks are related to the fact that they are often places to come in contact with nature. In both cross-sectional and longitudinal cohort studies, living near nature has consistently been associated with many physical, mental, and social health benefits, including reduced diabetes, improved depression, and improved risk of stress-related conditions such as heart disease [7]. Examples include lowering the risk of delivering a low-birthweight infant for pregnant women, reduced ADHD symptoms for children, and lower risk of myocardial infarction and heart failure [8,9]. For children, living in a neighborhood with little greenspace is predictive of psychiatric disease in adulthood [10]. Nature has also been associated with reduced mortality [11]. Another large body of literature demonstrates immediate physiologic and emotional improvements during and after nature visits [12]. Experimental and quasiexperimental studies have also provided evidence that neighborhood nature can improve health [13,14].

Today, clinicians across the nation are engaged in park and health partnerships, or park prescription programs, for the purpose of improving patient health [15]. Now more than 200 in number, these programs range in design from those supported by park districts with free access to parks, to those that integrate electronic records and referrals to parks nearest to people’s homes, to those supporting family access to parks with transportation or other facilitation [16]. Experimental studies evaluating this practice also are on the rise. To date, the few experimental studies on park prescription programs have shown moderate adherence [17], increased physical activity [18], improved quality of life [19], and improved resilience in children who received park prescriptions [20]. Park prescriptions for families demonstrated increased weekly park visits and lowered stress in parents [21]. Other nature-based therapies such as horticulture have shown biologic benefits such as reduced proinflammatory cytokines seen in elderly participants of a randomized trial [22]. In a randomized trial of park prescriptions in Indonesia, the numbers of healthy individuals 40 to 65 years old who spent time in parks, who participated in physical and recreational park activities, and who saw an increase in psychological quality of life were significantly greater in the park prescription group [23], although there was no difference in total physical activity between the two groups.

Because research shows that nature exposure may be most beneficial to low-income communities and may decrease health inequities [4], park prescription programs have been started in low-income communities [20]. We currently lack information on how poverty may be associated with park access in clinic patients that may be candidates for a park prescription and on how those patients may prioritize park access. As the site of an intervention to address social and economic stressors in pediatric clinical settings, we asked a sample of low-income families about their access to parks. During an in-person screening and case-management intervention targeting pediatric social needs, we asked low-income families about unmet social needs, including access to parks (defined in this paper as parks, playgrounds, or greenspaces). We further asked families to rank their top three unmet needs. Our hypothesis was that sociodemographic inequities would be reflected in self-reported access to parks. 

## 2. Methods

We administered a survey to adult caregivers of pediatric patients at a Federally Qualified Health Center (FQHC) and an urgent care clinic in the same health system located in an urban center serving a largely low-income population; 90% of the population served is on public insurance. Eligible participants were English-speaking or Spanish-speaking caregivers, 18 years or older, who were familiar with the child’s household environment and were living in the county where enrollment took place. Families seeking health care for a child with severe illness were excluded. Only one caregiver and child were enrolled per household. Surveys were administered by volunteers recruited from local universities who received eight hours of training covering intervention procedures; cultural accountability; community, hospital, and government social services resources; and motivational interviewing. They also received on-going, on-site training from supervisors, including observed volunteer–participant interactions with feedback for quality improvement from research staff. 

Survey data were collected electronically (on tablets); this procedure has been shown to result in more accurate assessments of social needs than the use of paper questionnaires [24]. Study data were collected and managed using Research Electronic Data Capture (REDCap) electronic data capture tools hosted at the University of California at San Francisco [25]. REDCap is a secure, web-based application designed to support data capture for research studies, providing (1) an intuitive interface for validated data entry, (2) audit trails for tracking data manipulation and export procedures, (3) automated export procedures for seamless data downloads to common statistical packages, and (4) procedures for importing data from external sources. All participants were encouraged to ask for technical assistance if needed at any point, and the electronic survey could be reinitiated after any discontinuation. Participants could view the survey in their preferred language and were given headsets so they could use audio assist with identical, prerecorded questions. Respondents were given the option to skip any items. Participants were issued a $5 gift card after survey completion. The survey instrument was available in English and Spanish.

All adult caregivers completed written informed consents, and additional consents for participation were obtained for children aged seven years or older. Study recruitment and follow-up took place on a predetermined timeline between 13 October 2013 and 27 August 2015 at safety-net hospitals where most patients are enrolled in Medicaid or lack health insurance coverage. 

Study protocols and materials were approved by the University of California, San Francisco, Committee on Human Research, and the Children’s Hospital and Research Center Oakland Institutional Review Board (UCSF study number: 13-11628). Further details about study protocol are specified elsewhere [26].

### 2.1. Measures

#### 2.1.1. Demographic Variables 

We collected data on the caregiver’s and their child’s ages, the caregiver’s and the child’s gender, the child’s race/ethnicity, family income, and caregiver’s education level. 

#### 2.1.2. Social Needs 

Caregivers endorsed (yes or no) any unmet needs that their families were currently experiencing, using a 14-item social and mental health needs screening questionnaire. Items included housing stability and habitability, food and income insecurity, child care and transportation needs, employment, legal concerns, medical insurance and other public health benefits enrollment, and concerns about any adult household member’s mental health. While no existing well-validated, multi-social-domain tools are being used in clinical settings, the original study team did publish findings on the acceptability of the social risk screening tool that was used as the basis for this work [25]. 

#### 2.1.3. Park Access 

We added a question about the need for access to parks (“access to greenspaces, parks, or playgrounds”). While this question has not been studied, self-reported park access has been associated more closely with the number of walking trips per month than objective accessibility, and subjective assessment of greenspace has been more closely correlated with walking than objective measures of access to greenspace [27]. After participants endorsed unmet needs, they were asked to rank their top three needs, which we considered their “prioritized” needs. 

### 2.2. Statistical Analysis

We tabulated descriptive statistics of the sample and calculated the number and percentage of caregivers who marked “yes” to each of the 14 social needs plus park access. We examined associations between access to parks (yes/no) with family demographic factors (age, gender, parent education, income level, race, ethnicity) using chi-squared or Fisher’s exact tests and t-tests for continuous variables. We examined associations between prioritizing outdoor space (yes/no) with other unmet needs using chi-squared or Fisher’s exact tests and t-tests for continuous variables. We then conducted a logistic regression analysis to examine the association between poverty (predictor) and park access (outcome). Potential confounders of age, gender, parent education, and race/ethnicity were chosen a priori based on research showing their association with both the predictor and the outcome. The resulting multivariable model was presented. 

All hypotheses were two-tailed and had 95% confidence intervals. Analyses were conducted using Stata SE, version 14SE, for Macintosh (Stata Corporation, College Station, TX, USA).

## 3. Results

The survey was completed by 890 consenting caregivers. The median age of their children was four years (IQR 1–8 years); 39.4% were non-Hispanic Black, 33.6% Hispanic White, and 7.5% Hispanic Black (Table 1). Caregivers were predominantly female (88.9%) with a mean age of 32.3 years; 72.7% had a high school degree or greater. The total number of unmet social needs at baseline ranged from 0–11 out of 14 total standard items, with a median of two (IQR 1–4) per family. Of the 890 families, 166 (19%) did not endorse any needs, while 183 (21%) reported four or more needs (not shown). Lack of park access was more frequent among families living below the poverty line (*p* = 0.005), among caregivers ages 25 to 44 years old (*p* = 0.013), and among caregivers with lower caregiver education level (*p* = 0.016) (Table 1). A child’s age, gender, or race/ethnicity was not significantly associated with lack of park access. 

Most participants did not lack access to parks. Access to parks was endorsed as an unmet need by 151 caregivers (17%) (Table 2). This is compared to 397 (45%) who endorsed “running out of food before you had money or food stamps to buy more”, or 381 participants (43%) who endorsed “not having enough money to pay your utility bills, like electric, gas, water, or phone”, 248 (28%) who endorsed “trouble finding a job”, 232 (26%) who endorsed “medical bills”, and 219 (25%) who endorsed “difficulty with housing”. Lacking access to parks was the sixth most commonly reported social need and was ranked just above not having health insurance (Table 2).

Of the 151 caregivers who reported access to parks as an unmet need, 33 (22%) prioritized it as one of their top three needs (Table 2). Prioritizing park access as an unmet need was associated with having less than a high school education (*p* = 0.006) and was inversely associated with access to other basic social needs such as food (*p* < 0.001), having money to pay utility bills (*p* < 0.001), having trouble finding a job (*p* < 0.001), medical bills (*p* < 0.001), and not having a place to live (*p* < 0.001) (Table 2).

The relationship between poverty level and park access (whether or not it was prioritized) was examined through a logistic regression analysis. Child age, child race/ethnicity, caregiver gender, caregiver age, and caregiver level of education less than high school were adjusted for Table 3. Those living at or below the poverty line were twice as likely to report a lack of access to parks compared to respondents living above the poverty line (OR 1.96, 95% CI 1.16–3.31) when controlling for other factors (Table 3). Being a high school graduate or college graduate was inversely correlated with lacking access to a park; caregivers with less than high school education had 71% higher odds (95% CI 1.17–2.49) of lacking park access than those who had graduated high school. Older caregivers had clinically insignificant (1%) lower odds of lacking access to a park (Table 3).

## 4. Discussion

While many families expressed that they had park access, poverty and lower parental education were significantly associated with access to parks being an unmet need. Families facing housing, food, and employment insecurity were less likely to prioritize park access.

Our finding that the lowest income patients were the ones least likely to have access to parks is important in the context of park prescription programs. Other research has had mixed findings on socioeconomic correlates of park access, with some finding that parks and recreational spaces are less available in low socioeconomic and high minority communities [28,29]. Alternatively, some studies have shown no association [30], or a higher availability of parks, recreational areas, or greenspace in low-income and high minority areas compared to higher-income and low minority areas [31]. Because our study evaluated self-reported assessment, it may capture perceived access. Our finding raises the important conundrum that the people who may be the hardest to reach through a park prescription program are those who also face the most health inequity. In essence, those who face the greatest health challenges and who may be served by a park prescription may be those who have the least access to parks.

Park access was less likely to be prioritized as an unmet need when families were also experiencing insecurity related to food, housing, utilities, or employment. In our other work, we have found that issues of time and money were most likely to be associated with who followed through with a park prescription [32]. We recommend that park prescription programs consider competing priorities in their design. An alternate strategy is to position park referrals not in competition with other priorities, but as a strategy to cope with other stressors. Indeed, the nature found in parks has been shown to buffer the effects of poverty on health outcomes in adults [4], housing insecurity among refugees [33], and the effects of stress on childhood adversity in children [19,34]. We suggest that park interventions seeking to buffer the negative effects of poverty on health outcomes be explicit in naming and accounting for inequity in access to greenspaces.

As park prescription programs expand across the US, our findings could have implications for clinical practice and health equity. To be maximally effective, clinical interventions targeting park use in low-income settings may need to be offered in conjunction with supports related to basic material needs like food, housing, and utilities. Indeed, some have raised the concern that the prescription model does not consider the relationships between socioeconomic barriers and time spent in nature [35], arguing that the disproportionate use and distribution of outdoor recreational spaces such as parks are rooted in racial segregation and social inequality [36]. 

Our study has three key limitations. First, the generalizability of our findings is limited by our small and geographically restricted sample. Access to parks may differ in other settings, although overall our results are consistent with other research that has shown that income predicts access to nature. Second, no validated patient-reported survey measures exist regarding time in nature, and we were not able to directly measure access to parks. Third, access to parks and nature are not necessarily the same thing. In the future, we will need to find a way to look at whether patients live in nature-deprived areas as separate from park exposure. Finally, the cross-sectional survey design prohibits causal inference.

## 5. Conclusions

Despite these limitations, our study sheds light on questions about park access, which may be of use to clinicians hoping to engage patients in active living through parks. Providers working with low-income families living in urban centers should recognize that poverty may be a common barrier to health and that this barrier is strongly associated with other social and economic obstacles to health promotion. More research is needed to understand how park access may influence the success of park prescription programs.

## Figures and Tables

**Table 1:** Participant characteristics and self-reported unmet need for parks during a primary care social determinants screen (*n* = 890).

Characteristics	Total	%	Unmet Need	(%)	*p*-Value
	*n*		*n*		
Child’s gender					0.054 b
Male	409		59	(14)	
Female	471	53	91	(19)	
Child’s age					0.511 b
0 to 4	545		89	(16)	
1 to 5	206		41	(20)	
6 to 12	71		12	(17)	
Child’s race and ethnicity					0.753 a
White non-Hispanic	34	−3.8	5	(15)	
Hispanic	255	−33.6	44	(17)	
Black non-Hispanic	287	−39.4	63	(18)	
Black Hispanic	54	−7.5	13	(24)	
Asian	35	−5	10	(29)	
Pacific Islander	6	−0.9	2	(33)	
Native American or Alaskan Native	6	−0.7	0	0	
Other	66	−9	14	(1)	
Caregiver gender					0.293 b
Male	95		20	(21)	
Female	765	−88.9	128	(17)	
Caregiver age					0.013 b
18 to 24	185		23	(12)	
25 to 34	352		78	(22)	
35 to 44	229		32	(14)	
45 and above	76		12	(16)	
Highest level of education					0.016 b
Less than high school	233	−27.3	54	(23)	
High school graduate	478	−56	74	(15)	
College or graduate school	142	−16.7	19	(13)	
Federal poverty level					0.005 b
Above federal poverty level	226		24	(11)	
At or below federal poverty level	528		100	(19)	
Number of social needs (out of 14) median and IQR	2 (1–4)				

a. *p*-value calculated using Fisher’s exact test; b. *p*-value calculated using chi-squared test.

**Table 2:** Social needs at baseline from a standardized 14-item social and mental health needs screening questionnaire, and percent of those respondents that prioritized access to parks as a top-three social need (*n* = 890).

Item	*n*	(% of Total)	Prioritized Access to Parks and Playgrounds	*p*-value
Running out of food before you had money or food stamps to buy more	397	(45)	7	<0.001 *
Not having enough money to pay your utility bills, such as electric, gas, water, or phone	381	(43)	10	<0.001 *
Trouble finding a job	248	(28)	4	<0.001 **
Medical bills	232	(26)	3	<0.001 **
Not having a place to live for example, concerns about eviction, foreclosure, staying with friends/family, and current homelessness	219	(25)	2	<0.001 **
Lack of access to green spaces/parks/playgrounds	151	(17)	33	n/a
No health insurance	150	(17)	3	<0.001 **
Unhealthy living environments, for example, problems such as mold, insects, rats or mice, excess trash	147	(17)	6	
Being cut off or denied from programs that provide income supports to your family, such as CalFresh (food stamps) or CalWorks (welfare)	143	(16)	4	
No primary care or regular general doctor	116	(13)	3	
Other concerns with your housing	114	(13)	4	
Disability-related impairment interfering with ability to work	96	(11)	3	
Accessing mental health care for you or another caregiver in your household	63	(7)	3	
Problems with a current or former job, such as unpaid wages, worker’s compensation, wrongful termination, discrimination or harassment, or needing unemployment insurance	53	(6)	0	<0.001 **
Concerns about pregnancy-related work benefits	19	(2)	0	

* chi-squared test; ** Fisher’s exact test.

**Table 3:** Predictors of self-reported unmet need for access to parks during a primary care social determinants screen (*n* = 822)^a^.

Parameter	Odds Ratio	95% Confidence Interval	*p*-Value
Family living at or below the federal poverty level	1.96	(1.16–3.31)	0.012
Child’s gender female	1.31	(0.87–1.98)	0.183
Child’s age	1.02	(0.98–1.08)	0.264
Child’s race and ethnicity			
White (Comparison)	–	–	–
Hispanic White	0.7	(0.23–2.10)	0.527
Black	1.35	(0.72–2.55)	0.35
Hispanic Black	1.03	(0.30–3.49)	0.961
Asian	1.59	(0.423–6.01)	0.49
Pacific Islander	0.51	(0.05–5.63)	0.584
Other	1.05	(0.31–3.55)	0.942
Caregiver’s gender female	0.53	(0.98–1.08)	0.228
Caregiver’s age	0.99	(0.29–0.97)	0.04
Caregiver’s level of education less than high school	1.71	(1.17–2.49)	0.005
Family living at or below the federal poverty level	1.96	(1.16–3.31)	0.012

a. Multivariable regression analysis with endorsing unmet need for outdoor spaces as outcome and listed variables as predictors. – reference value.
